# A Novel Application of Non-Negative Matrix Factorization to the Prediction of the Health Status of Undocumented Immigrants

**DOI:** 10.1089/heq.2021.0079

**Published:** 2021-12-13

**Authors:** Jason Li, James Wells, Chenli Yang, Xiaodan Wang, Yihan Lin, You Lyu, Yan Li

**Affiliations:** ^1^ Austin College, Sherman, Texas, USA.; ^2^ Department of Physiology, Tulane University, New Orleans, Louisiana, USA.; ^3^ Kunming Children's Hospital, Kunming, Yunnan, China.; ^4^ Yunnan Institute for Drug Abuse, Kunming, Yunnan, China.; ^5^ HKU-Pasteur Research Pole, Hong Kong, China.; ^6^ Omni Medical Center, Plano, Texas, USA.

**Keywords:** undocumented immigrants, machine learning, cardiovascular risk

## Abstract

**Introduction:** Undocumented immigrants (UIs) in the United States are less likely to be able to afford health insurance. As a result, UIs often lack family doctors and are rarely involved in annual screening programs, which makes estimating their health status remarkably challenging. This is especially true if the laboratory results from limited screening programs fail to provide sufficient clinical information.

**Methods:** To address this issue, we have developed a machine learning model based on the non-negative matrix factorization technique. The data set we used for model training and testing was obtained from the 2004 cost-free hepatitis B screening program at the Omni Health Center located in Plano, Texas. Total 300 people were involved, with 199 identified as UIs.

**Results:** People in the UIs group have higher cholesterol (219.6 mg/dL, *p*=0.038) and triglycerides (173.2 mg/dL, *p*=0.03) level. They also have a lower hepatitis B vaccination rate (38%, *p*=0.0247). No significant difference in hepatitis B^(+)^ was found (*p*=0.8823). Using 16 individual clinical measurements as training features, our newly developed model has a 67.56% accuracy in predicting the ratio of cholesterol to high-density lipoprotein; in addition, this newly developed model performs 9.1% better than a comparable multiclass logistic regression model.

**Conclusions:** Elderly UIs have poorer health status compared with permanent residents and citizens in the United States. Our newly developed machine learning model demonstrates a powerful support tool for designing health intervention programs that target UIs in the United States.

## Introduction

The estimated total number of immigrants without legal status, or undocumented immigrants (UIs), in the United States has remained relatively stable since at least the early 2000s,^1,2^ consisting of roughly 4% of the U.S. population, or 12 million individuals. The specific demographic distribution varies greatly from state to state, with California, Texas, and Florida having the largest number of UIs.^3^ Furthermore, there is a great amount of variation in the inclusivity of state-specific immigrant policies, especially as it relates to UIs, which results in large differences in terms of the medical burden that individual state-based health care systems face.^4^ In Texas, a state with roughly 1.7 million UIs, it is estimated that 32% of UIs live below the poverty line and 64% of UIs are uninsured.^5^

Comparatively, of the >2.6 million UIs in California, a state with one of the highest UI insurance rates, an estimated 40% are uninsured.^6^ These socioeconomic challenges are further compounded by numerous other barriers to care, including discrimination, linguistic barriers, and fears of deportation. In addition, these barriers often have an especially high impact on elderly UIs' access to health care, who are already at greater medical risk in regard to most chronic conditions.^7^ All of these factors, and the frequent local administration of accessible health care, make the collection of personalized data for population-based monitoring and control of both acute and chronic diseases in UI populations extremely difficult.

As a result of the aforementioned barriers that many UIs face in seeking health care, UIs frequently rely on safety nets to address health care needs such as infectious diseases, mental health issues, prenatal care, and childbirth-related care.^8^ The frequent reliance on safety net resources often results in delayed primary care, acute care overutilization, and ultimately poor health outcomes due to already limited operating resources becoming overwhelmed. Ultimately, it currently falls to state and local governments to attempt to enact measures that extend health care services to UIs, and they often must do so while facing explicit federal restrictions on funding.^9^

Nevertheless, even in major metropolitan areas that have implemented strategies and interventions to extend health care services to UIs, such as New York City, Atlanta, Dallas, Houston, and Chicago, federal policy and rhetoric have continued to limit the utilization of these services by UIs.^10^ In summary, this combination of various socioeconomic and political factors represents a huge challenge in providing quality health care to UIs, and in implementing health-related medical research relating to UI health in the United States.

As a result of the already limited interactions that UIs have with health care providers, there is considerable potential value in utilizing pre-existing clinical information to identify individuals who are at high risk for specific chronic conditions. Recently, data-driven machine learning algorithms have been used to predict specific health outcomes or potential pathophysiologies; examples of these include the determination of intensive care unit mortality risk and the prediction of abnormal cardiac mechanics.^11,12^ These modeling techniques facilitate the discovery of otherwise indiscernible relationships in complex multivariate problem spaces.

In this article, we describe the development of a new statistical model using the representation learning technique non-negative matrix factorization (NMF)^13^ and 16 clinic measurements to perform predictive modeling of the ratio of cholesterol to high-density lipoprotein (HDL) and the resulting personal health status. The new model described in this study presents an efficient way to handle the medical data generated from community-based primary care centers and offers a possible method for improving the quality and efficiency of UI health care delivery despite the unique barriers that this population currently faces.

## Methods

### Data collection

The free hepatitis-B screening program was conducted from May to June 2004 at Omni Health Center in Plano, Texas. Overall, this screening program involved 300 people. Patient-level variables that were collected included contact information, age, gender, race, weight, marital status, insurance condition, emergency contact(s), and social security number (SSN). Of note, patients who enrolled in the screening process without providing their SSN would be considered as UI.^14^ The molecular-level parameters that were tested included fasting glucose (GLU), cholesterol (CHOL), triglycerides (TG), HDL, low-density lipoprotein (LDL), thyroid-stimulating hormone (TSH), prostate-specific antigen (PSA), hepatitis B surface antigen (HBsAg), and hepatitis B surface antibody (HBsAb).

All the blood samples were sent to *Bio-Reference Laboratories, Inc*., in Elmwood Park, NJ for analysis and laboratory report generation. Four nurses were collectively responsible for the data collection, blood sample preparation, and follow-up process. Three physicians were assigned to evaluate the final reports and identify the necessary follow-up.

### Model development

To build our data-driven machine learning model, we first combined the patient-level and molecular-level data to obtain the target matrix, *X*. For this application regarding the health information of UIs, NMF has at least one major advantage over traditional multivariable regression models; that NMF is more easily adapted to missing input information, which results from the difficulties in gathering this health information discussed in the previous sections.^15^ In terms of how NMF is applied, this method first blindly decomposes the target matrix *X* into two non-negative matrices, *U* and *V*; then it iteratively solves the objective function $\min_{U,V}\frac{1}{2}\left||X-UV\right|{|}_{F}^{2}$ to find the minimum, where $\left||\cdot \right|{|}_{F}$ denotes the matrix Frobenius norm; finally, the training process was completed once the iterations met the convergence criteria.

In this study, we propose a new scheme name: the orthogonal gradient NMF method (OGNMF) to improve solver efficiency. Under this new scheme, a total of 16 features have been implemented to predict the missing values on the final laboratory report for the 300 samples. These clinical features including have a social security number (SSN^(+)^) or not having a social security number (SSN^(−)^), patient account number, gender, age, race, TSH, PSA, HBsAb, HBsAg, GLU, CHOL, TG, HDL, LDL, and HDL/CHOL, which were transformed into numerical values for model development. Depending on the percentage of HDL and ratio of CHOL to HDL (Table 1), four levels of risk were used as reference values when applying OGNMF to the sample data.

**Table 1.** Percentage of High-Density Lipoprotein and the Ratio of Cholesterol to High-Density Lipoprotein Reflects the Health Status of the Screened Patient

HDL (%)	CHOL/HDL		Risk level
Male and female	Male	Female	
>25	<4.2	<3.9	Below-average risk
15–25	4.2–7.3	3.9–5.7	Average risk
15-9	7.4–11.5	5.8–9.0	Above-average risk (moderate)
<9	>11.5	>9.0	Above-average risk (high)

HDL, high-density lipoprotein.

Related details on how OGNMF was developed are given in Supplementary Data S1. The statistical analysis on the variables from the blood tests was conducted in R 3.6 with RStudio and the OGNMF-based machine learning model was implemented using the Matlab 2017b environment.

## Results

This study recruited 300 people with ages ranging from 16 to 93 years old. Overall, the UIs made up 66.3% (199/300) of screened participants. The details of blood test results were stratified by the SSN status and are listed in Table 2. The average age of the SSN^(+)^ group during the screening was 48.7 years old, whereas the average of the SSN^(−)^ group was 53.9 years old (*p*=0.00117). Patients in the SSN^(−)^ group had higher cholesterol (219.6 mg/dL compared with 208.9 mg/dL, *p*=0.038) and TG (173.2 mg/dL compared with 148.4 mg/dL, *p*=0.03) than people in the SSN^(+)^ group. Patients in the SSN^(+)^ group had a lower hepatitis B vaccination rate than people in the SSN^(−)^ group (38% compared with 52%, *p*=0.0247).

**Table 2.** Comparison of health status with selected variables for the patient with SSN^(+)^ and SSN^(−)^

Characteristic		SSN^(−)^					SSN^(+)^			
	Sample size, *n* (%)	Mean	SD	Min.	Max.	Sample size, *n* (%)	Mean	S.D.	Min.	Max.
Age (years)	N.A.	53.9	14.2	16	93	N.A.	48.7	12	20	75
FG (mg/dL)		93.2	24.5	47	226		88.2	18.3	58	194
CHOL (mg/dL)^a^		219.6	41.1	140	354		208.9	39	129	341
TG (mg/dL^)b^		173.2	91.8	46	663		148.4	104.2	14	801
CHOL % (CHOL/HDL)		3.9	1.1	1.8	8.6		3.8	1.5	1.9	12.7
LDL (mg/dL)		126.8	33.3	55	238		120.1	37	0.9	214
TSH (mIU/L)		2.1	1.2	0.1	6.37		2.3	1.5	0.3	12.8
HBsAb^(+)^, *n* (%)	104 (52)	N.A.				39 (38)	N.A.			
HBsAg^(+)^, *n* (%)	18 (9)					8 (8)				

a,b Two-sample *t*-test, with *p*-value=0.0388, 0.03, respectively.

CHOL, cholesterol; CHOL% (CHOL/HDL), total-cholesterol-to-HDL cholesterol ratio; FG, fasting glucose; HBsAb, hepatitis B surface antibody; HBsAg, hepatitis B surface antigen; LDL, low-density lipoprotein; SSN, social security number; Min., minimum; Max., maximum; N.A., not applicable; SD, standard deviation; TG, triglycerides; TSH, thyroid-stimulating hormone.

Overall, 8% of people in the SSN^(+)^ group and 9% of people in the SSN^(−)^ group were hepatitis B positive (*p*=0.8823). We did not observe any significant health status disparities between the SSN^(+)^ and SSN^(−)^ groups, in terms of TSH, fasting glucose, and so on. The PSA screening test was conducted on 43 patients, and none of them had PSA levels >4.2 ng/mL (Supplementary Data).

**FIG. 1.**
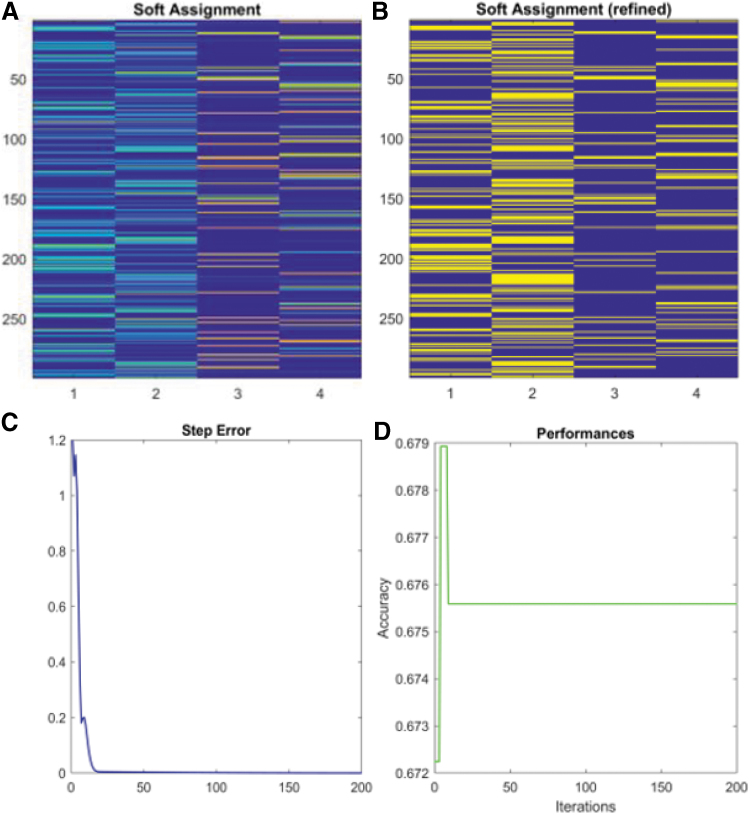
We developed a machine-learning model (*ɛ*=1e^−5^) with 16 measured variables from laboratory reports. **(A)** Soft assignment method determined initial data distribution (represented by the width of the bands) at different levels of health risk. **(B)** More smooth bands were generated by the OGNMF scheme in every iteration. **(C)** Step error decreases and reaches a plateau after 10 iterations. **(D)** Predictive accuracy reaches 67.59% after 10 iterations and remains stable. NMF, non-negative matrix factorization; OGNMF, orthogonal gradient NMF method.

To test the performance of the newly developed model, we selected ɛ equal to 1e^−2^, 1e^−3^, 1e^−4^, and 1e^−5^ as the four stopping criteria. Based on the ratio of CHOL to HDL, we assigned the personal-level health statuses of 1, 2, 3, and 4 in response to below-average risk, average risk, above-average risk (moderate), and above-average risk (high), respectively^16^ (Table 1). When ɛ was equal to 1e^−5^, we applied the soft-assignment method to cluster the ratio of CHOL to HDL and obtained the scaled plot for all patient samples with different risk levels (Fig. 1A). Since the original CHOL/HDL ratio is recoded by the categorical numbers 1–4, the width of each colored band in every column represents how closely the original data were to the cutoff value of each risk category.

After iteration, OGNMF produces more distributed bands that represent the patient's data at different risk levels (Fig. 1B). The step error generated during each iteration decreases dramatically at the beginning then reaches a plateau after 10 steps (Fig. 1C). Simultaneously, the prediction accuracy reaches 67.59% when the step error reaches a steady state (Fig. 1D). Numerical data associated with other epsilons (*ɛ*) are given in the Supplementary Data. As cross-validation, we randomly selected patients from the same data set and calculated the CHOL/HDL ratio by using a multiclass logistic regression (MLR) method. The average prediction efficiency of this MLR was equal to 61.59%, which is 9.7% less accurate than our newly developed OGNMF-based model.

## Discussion

As an extremely vulnerable population within the United States, UIs do not have access to most of the medical insurance options that other legal residents have access to. In addition, the federal budget, which supports the operation of safety net hospitals that frequently are the only health care option for UIs, is extraordinarily restricted and often limited to specific medical cases.

The primary avenue that UIs have in terms of federal funding is Emergency Medicaid eligibility, which is the only exception to the exclusion of UIs from Medicaid coverage.^17^ The major Emergency Medicaid expenditures among UIs are acute workplace injury, childbirth, and pregnancy-related complications, but expenditures related to chronic conditions, such as chronic renal failure, cerebrovascular disease, and heart disease, are increasing.^8,17^

When UIs can receive health care or seek out emergency care services, often only specific disease-related clinic parameters are tested and recorded; inadequately studied clinical parameters that are potentially pathophysiologically relevant, such as thyroid function in heart failure patients, are often uncaptured.^18^ Recent history has provided a clear case study illustrating how such limited medical records from UI populations can impede public health officials' ability to deal with infectious disease outbreaks, specifically the COVID-19 pandemic, in various American UI communities.^19^

To demonstrate the potential that machine learning techniques have in helping to address these gaps in UI clinical health care data, we developed a new OGNMF-based predictive model described in the previous sections. This new model has a fast convergence rate and better computational accuracy when using a data set that contains the medical screening information from UIs compared with models developed using other techniques. In addition, concerning other machine-learning models,^15^ this is the first machine learning model designed for assisting in the analysis of the health statuses of UIs.

It is worth noting that, we did not observe an association between health insurance and health outcomes in UIs or legal residents/citizens. One potential cause of this lack of association could be that health insurance information was missing for many of the participants. Only 11% of screening patients filled out their health insurance and none of those who filled out this information came from the UI group (Table 3). Meanwhile, Asian participants made up a large percentage of the sample population, which could have resulted in selection bias when developing our statistical model.

**Table 3.** Descriptive Statistics of Demographic Feature on Total Screening Patients (*n*=300)

Variable name, *n* (%)	Undocumented immigrants	Legal residents/citizen
Sample size	199 (66.3)	101 (33.7)
Covered by health insurance	0	33 (11)
Male	106 (53)	58 (57)
Female	93 (47)	43 (43)
Chinese Americans	132 (66)	76 (75)
Other Asian Americans	52 (26)	12 (12)
Other races	15 (8)	13 (13)

To demonstrate that the accuracy of the newly developed OGNMF model was not significantly affected by the missing values in the selected features, we treated all data in the blood tests as independent variables and cross-validated the model using published physical screening data from the Wanfang Data, a database that provides a wide range of Chinese data from Chinese studies, and from medical and scientific research. The OGNMF-based model did not show better prediction accuracy when trained using the data published in the Wanfang Database.^20^

In terms of the next steps to improve our model, we would like to work on quantitatively defining the intercorrelated variables such as age and TSH, cholesterol, and the CHOL/HDL ratio to further improve the model's predictive accuracy and specificity.

## Conclusions

UIs in the United States are less likely to be covered by health insurance and receive consistent physical screening tests. Our newly developed statistical model offers a potentially strong support tool for health providers to address the sampling issue of vulnerable minority groups.

## Supplementary Material

Supplemental data

## Data Availability

The de-identified patient's blood test data are given upon request.

## Abbreviations Used

CHOL: cholesterol
GLU: glucose
HBsAb: hepatitis B surface antibody
HBsAg: hepatitis B surface antigen
HDL: high-density lipoprotein
LDL: low-density lipoprotein
MLR: multiclass logistic regression
NMF: non-negative matrix factorization
OGNMF: orthogonal gradient NMF method
PSA: prostate-specific antigen
SSN: social security number
TG: triglycerides
TSH: thyroid-stimulating hormone
UIs: undocumented immigrants
