# Supplementary material for: A Novel Application of Non-Negative Matrix Factorization to the Prediction of the Health Status of Undocumented Immigrants
Source: Health Equity. 2021 Dec 13;5(1):834–9. doi: 10.1089/heq.2021.0079 (PMC8742291; doi:10.1089/heq.2021.0079)
Supplement: Supplemental data [file Suppl_Data.docx]

**Supplementary Material**

**Non-negative Matrix Factorization Method**

Specifically, given the data matrix $\boldsymbol{X}=\left[ \boldsymbol{x}_{1},\boldsymbol{x}_{2},\cdots,\boldsymbol{x}_{n} \right]\in\mathfrak{R}^{d\times n}$, where *n* is the total sample size (*n* = 300), *d* is the selected features (*d* = 16), and each column of $\boldsymbol{X}$ is a sample vector. The key idea of NMF is to optimally determine two non-negative matrices $\boldsymbol{U}=\left[ u_{ij} \right]\in\mathfrak{R}^{d\times k}$ and $\boldsymbol{V}=\left[ v_{ij} \right]\in\mathfrak{R}^{k\times n}$, which can minimize the following objective function:

|  | $\min_{\boldsymbol{U},\boldsymbol{V}} \frac{1}{2}\left\Vert\boldsymbol{X-UV} \right\Vert_{F}^{2}$ , | (1) |
| --- | --- | --- |

where $\left\| \cdot\right\|_{F}$ denotes the matrix Frobenius norm, and $k\leq min\left\{ d,n \right\}$. Since equation (1) belongs to a non-convex minimization problem, it is impractical to obtain the optimal solution. Fortunately, the block coordinate descent method can obtain a local solution of equation (1). Given an initial $\boldsymbol{U}^{1}\geq\mathbf{0}$, the block coordinate descent method iteratively computes

|  | $\boldsymbol{V}^{t+1}=arg\min_{\boldsymbol{V}\geq\mathbf{0}} \frac{1}{2}\left\Vert\boldsymbol{X}-\boldsymbol{U}^{t}\boldsymbol{V} \right\Vert_{F}^{2}$ | (2) |
| --- | --- | --- |

and

|  | $\boldsymbol{U}^{t+1}=arg\min_{\boldsymbol{U}\geq\mathbf{0}} \frac{1}{2}\left\Vert\boldsymbol{X}^{T}-\left( \boldsymbol{V}^{t+1} \right)^{T}\boldsymbol{U}^{T} \right\Vert_{F}^{2}$ | (3) |
| --- | --- | --- |

until convergence, where $t$ is the iteration counter. Most existing NMF solvers require special implementations to solve equations (2) and (3), and the optimization strategies for minimization are varied. Since equations (2) and (3) are symmetric, we proposed a new scheme: the orthogonal gradient method to improve the NMF solver efficiency.

| Algorithm |
| --- |
| Input: $\boldsymbol{X}=\left[ \boldsymbol{x}_{1},\boldsymbol{x}_{2},\cdots,\boldsymbol{x}_{n} \right]\in\mathfrak{R}^{d\times n}, 1\leq k\leq min\left\{ d,n \right\}$  Output: $\boldsymbol{U}=\left[ u_{ij} \right]\in\mathfrak{R}^{d\times k}$ and $\boldsymbol{V}=\left[ v_{ij} \right]\in\mathfrak{R}^{k\times n}$  1: Initialize $\boldsymbol{U}^{0}\boldsymbol{\geq0}$, and $\boldsymbol{V}^{0}\boldsymbol{\geq0}$, $a_{0}=b_{0}=1$, $A=\left\Vert\left( \boldsymbol{U}^{t} \right)^{T}\boldsymbol{U}^{t} \right\Vert_{2}$, $t=1$  2: Repeat  3: Update $\boldsymbol{V}^{t}\boldsymbol{=}P\left( \boldsymbol{V}^{t}\boldsymbol{-}\frac{1}{A}\nabla_{\boldsymbol{V}}\left\Vert\boldsymbol{X-}\boldsymbol{U}^{t}\boldsymbol{V} \right\Vert_{F}^{2} \right)$  4: Update $a_{t+1}=\frac{1+\sqrt{4a_{t}^{2}+1}}{2}$,  5: Update $\boldsymbol{V}^{t+1}\boldsymbol{=}\boldsymbol{V}^{t}\boldsymbol{+}\frac{a_{t}-1}{a_{t+1}}\left( \boldsymbol{V}^{t}\boldsymbol{-}\boldsymbol{V}^{t-1} \right)$  6: Update $\boldsymbol{U}^{t}\boldsymbol{=}P\left( \boldsymbol{U}^{t}\boldsymbol{-}\frac{1}{A}\nabla_{\boldsymbol{U}}\left\Vert\boldsymbol{X}^{T}\boldsymbol{-}\left( \boldsymbol{V}^{t+1} \right)^{T}\boldsymbol{U}^{T} \right\Vert_{F}^{2} \right)$  7: Update $b_{t+1}=\frac{1+\sqrt{4b_{t}^{2}+1}}{2}$,  8: Update $\boldsymbol{U}^{t+1}\boldsymbol{=}\boldsymbol{U}^{t}\boldsymbol{+}\frac{b_{t}-1}{b_{t+1}}\left( \boldsymbol{U}^{t}\boldsymbol{-}\boldsymbol{U}^{t-1} \right)$  9: $t\leftarrow t+1$  10: until the stopping criterion $\left\Vert\boldsymbol{V}^{t+1}\boldsymbol{-}\boldsymbol{V}^{t} \right\Vert_{F}^{2}<\varepsilon$ and $\left\Vert\boldsymbol{U}^{t+1}\boldsymbol{-}\boldsymbol{U}^{t} \right\Vert_{F}^{2}<\varepsilon$ is satisfied  11: Output: $\boldsymbol{U=}\boldsymbol{U}^{t+1}$, and $\boldsymbol{V=}\boldsymbol{V}^{t+1}$ |

where $P\left( \cdot\right)$ is the operator that projects all the negative entries to zero, and $\varepsilon>0$ is a pre-defined threshold.
